# Unexpected consequences of a drier world: evidence that delay in late summer rains biases the population sex ratio of an insect

**DOI:** 10.1098/rsos.150198

**Published:** 2015-09-02

**Authors:** Raul Bonal, Marisa Hernández, Josep M. Espelta, Alberto Muñoz, José M. Aparicio

**Affiliations:** 1 Forest Research Group, INDEHESA, University of Extremadura, Plasencia, Spain; 2 Grupo de Investigación de la Biodiversidad Genética y Cultural, Instituto de Investigación en Recursos Cinegéticos CSIC-UCLM-JCCM, Ciudad Real, Spain; 3 CREAF, Cerdanyola del Vallès, Catalonia 01893, Spain; 4 Departamento de Didáctica de la Ciencias Experimentales, Facultad de Educación, Universidad Complutense de Madrid, Madrid, Spain

**Keywords:** climate change, drought, insects, sex ratio

## Abstract

The complexity of animal life histories makes it difficult to predict the consequences of climate change on their populations. In this paper, we show, for the first time, that longer summer drought episodes, such as those predicted for the dry Mediterranean region under climate change, may bias insect population sex ratio. Many Mediterranean organisms, like the weevil *Curculio elephas*, become active again after summer drought. This insect depends on late summer rainfall to soften the soil and allow adult emergence from their underground refuges. We found that, as in many protandric species, more *C. elephas* females emerged later in the season. Male emergence timing was on average earlier and also more dependent on the beginning of late summer rainfall. When these rains were delayed, the observed weevil sex ratio was biased towards females. So far, the effects of global warming on animal sex ratios has been reported for temperature-dependent sex determination in reptiles. Our results show that rainfall timing can also bias the sex ratio in an insect, and highlight the need for keeping a phenological perspective to predict the consequences of climate change. We must consider not just the magnitude of the predicted changes in temperature and rainfall but also the effects of their timing.

## 1. Introduction

Climate change models predict longer and drier summers in the Mediterranean region [1,2]. Longer droughts will delay late summer/early autumn precipitations, which are critical to reactivate the life cycles of many species [3]. In this paper, we show, for the first time, that rain delay can have unexpected consequences on the demography of insect populations, biasing sex ratio.

In animals, females control primary sex ratio through resource allocation to male and female progeny when the fitness benefits of producing sons or daughters differ. This ability to control sex ratio is adaptive, as the fitness benefits of producing one sex or the other may depend on factors like female body condition or food availability [4]. Nonetheless, sex ratio sometimes escapes from females' control—i.e. temperature during embryonic development determines offspring sex in some reptiles [5] and fish [6]. This temperature dependence has introduced the risk of sex ratio alteration into the climate change debate: in reptiles, for example, biased offspring sex ratio unbalances the proportion of adult males and females, increasing matelessness and decreasing the effective population size [7].

Other than in many reptile species that have temperature-dependent sex determination [5,7], we know little about the effects of the climate on sex ratio. The primary sex ratio is the ratio at fertilization, secondary sex ratio the ratio at time of birth or hatching and tertiary sex ratio the ratio of the mature individuals in a population. The proportion of adult males and females in a population (i.e. tertiary sex ratio) does not thus depend exclusively on primary and secondary sex ratios, as a certain sex may suffer a higher mortality during development [8,9]. Tertiary sex ratio could be affected by climate change if one of the sexes were more vulnerable to the forecasted alterations. In the case of insects, for example, there are many species in which the onset of adult activity after diapause differs between sexes. In some cases, females are active before males (protogynous species), in others more males start their activity before females (protandrous species) [10]. Thus, the consequences of climate changes involving alterations in the timing of rainfall and/or extreme temperatures could differ between sexes.

We hypothesize that the longer droughts predicted by climate change models for dry Mediterranean areas could provoke a sex-biased mortality in insects like the acorn weevil *Curculio elephas* (Coleoptera: Curculionidae; Gyllenhal, 1836). *Curculio elephas* larvae overwinter in underground refuges and pupate shortly before emerging as adults from late summer to early autumn [11,12]. During the summer drought very little or no rain at all falls and the soil is dry and hard. According to our previous data, adult weevils are ready to emerge from the first week of September onwards [13]. They depend on the precipitation accumulated in the previous two weeks to soften the soil, as otherwise they cannot dig their way out from their underground refuges and may die [13,14]. These late summer storms are characteristic of dry Mediterranean areas, although their precise timing is variable [3]. If, as reported in other areas, males emerge earlier than females [14], delayed late summer rainfall (i.e. longer droughts) could constrain male emergence more strongly than female emergence and the population sex ratio might become female-biased.

For 5 years, we monitored a population of *C. elephas* to assess the patterns of emergence of males and females, their inter-annual variability and their dependence on the rainfall timing. We specifically assessed: (i) whether the number of males and females collected in emergence traps changed throughout the emergence period; (ii) to what extent the date of the beginning of late summer rains conditioned the emergence dates of males and females; and (iii) whether sex ratio was biased towards females in those years in which late summer rains were delayed.

## 2. Material and methods

### 2.1 Study area and species

We carried out the study in the locality of Huecas, province of Toledo, Central Spain, 40°00^′^ N, 04°11^′^ W. The climate is dry Mediterranean, with mean annual precipitation of just 365 l m^−2^, concentrated in spring and autumn. This climate is characterized by a severe summer drought: temperatures may reach 40°C and precipitation is extremely scarce or completely absent. July and August are the warmest and driest months: the rainfall recorded in these two months altogether accounts for less than 5% of the annual precipitation. The historical records of the closest meteorological station (from 1920 to 2012 both included) showed that August precipitation did not reach 1 l m^−2^ in 44 of those 93 years (in 17 years no rain was recorded). Summer drought ends with late August–September storms, these rains are predictable in a broad sense but their beginning is very variable and may show inter-annual differences of more than 30 days.

The study area covers a surface of 9 km^2^ and is a combination of open grasslands and cereal fields with scattered holm oaks, *Quercus ilex* [15]. There are isolated holm oaks and small forest plots within the cropland matrix. In the forest plots, tree density ranges from 20 to 50 trees per hectare, whereas in the case of isolated trees, the distance between oaks can range from 40 m to more than 2 km.

*Curculio elephas* is a specialist insect that feeds on acorns and chestnuts [11,12]. In Central Spain, it is the principal pre-dispersal predator of holm oak acorns and may attack more than half of the crop [11]. *Curculio elephas* larvae overwinter in underground earth-cells [13,14] at a depth of between 20 and 40 cm (R.B. 2006, personal observation); pupation takes place shortly before emerging as adults. Adults are ready to emerge from the first week of September onwards [13]; however, they need to dig their way up to the surface so dry, hard soil at critical times may reduce emergence rates. During the summer drought, the soil is extremely dry and hard, and late summer rains soften the soil and facilitate adult emergence [13,14]. Our previous data show that adult weevils start emerging one week after the first rainfall of late summer [13]. After emergence, adult weevils climb up the oak trunks; movement between trees is infrequent, especially if trees are scattered [16]. After climbing to the tree foliage, weevils mate and females oviposit into the acorns. Infested acorns are dropped prematurely and the larvae within them feed on the cotyledons until they finish their development. Then, larvae drill an exit hole through the seed coat, leave the acorn and immediately after that they bury themselves to overwinter underground [12,13]. Larvae will remain underground from 1 to 3 years, as females produce single broods consisting of offspring that have the potential to emerge over a 3-year period [14].

### 2.2 Field sampling

We randomly selected 25 focal oak trees to monitor *C. elephas* emergence during 5 years, from 2008 to 2012. Emergence traps were set up in July, before the onset of adult emergence. Each trap consisted of a mosquito net attached to the tree trunk with an inverted cone with a closed bottle on the top. The traps were not baited, weevils climbed up the trunk after leaving their underground cells and eventually marched into the net, which led them directly to the top bottle where they were trapped [15]. Traps covered the trunks partially and were placed close to the ground to capture the weevils climbing up after emergence—immigrants from other trees are more likely to arrive flying or marching onto the canopies. The number of traps placed at each tree ranged from one to three and was proportional to the canopy surface. Tree surfaces were calculated on the basis of three random measures of the diameter of their canopy, considering trees to be roughly circular [12]. Traps were checked and emptied every 3 days from 15 August to 15 December and the weevils were collected alive. At each visit, the number of adults at each trap was recorded and all of them were sexed, as female morphology is unmistakeable due to their extremely long rostrum [11,15]. Adults were then taken to the laboratory and their body size (i.e. elytra length) measured to the nearest 0.001 mm under a microscope.

Daily precipitation data came from the local meteorological station of Toledo (39°53^′^ N, 04°02^′^ W), which is 18 km from the study site, and rainfall was recorded on a daily basis. The area between the meteorological station and the study site is mostly flat. This minimizes differences between both sites because there are no mountains that might change the rainfall patterns. For the purpose of our analyses, we calculated the weekly precipitation starting from the second half of the summer: from week 1 (4–10 August) to week 12 (20–26 October).

The authorities of Huecas Town Council (province of Toledo, Spain) gave permission to carry out the study. Local landowners granted access to private fields.

### 2.3 Data analysis

In all analyses, the sampling unit was the trap and ‘tree identity’ was included as a random factor to control for potential local effects. We performed linear mixed models or generalized linear mixed models (GLMMs) depending, respectively, on whether the dependent variable was normally distributed or not.

We performed two GLMMs to test the effect of the ‘total amount of rain’ on the total number of adult weevils trapped each year (dependent variable with a Poisson distribution). The total amount of rain was the precipitation recorded in the period comprising between two weeks before the first and the last weevil emerged. We used this time lapse because our previous data showed that the number of weevils emerging in a certain week was correlated with the rain recorded one week [12] or even two weeks before.

We pooled the data of the five study years and used two GLMMs to assess whether the number of males and females (dependent variables, Poisson distributions) collected in the emergence traps changed from week to week along the emergence period. Another GLMM stating a binomial error was performed to analyse whether the sex ratio (male versus female counts) recorded in the traps changed along the emergence period too.

We carried out a linear mixed model to analyse whether adult weevil emergence date changed between years according to the date of the beginning of late summer rainfall and whether the effect of late summer rainfall differed between sexes. We considered the date of the beginning of late summer rains as the date after 4 August with a daily precipitation of at least 1 l m^−2^, which is enough to promote weevil emergence [13]. This time lapse is conservative, as it is usually from late August on when storms are more likely. Also, any rain before 4 August will not have any effect on weevil emergence, as it depends on the rain accumulated in the previous one or two weeks and adult weevils are not ready to emerge until the first week of September [13].

To test whether population sex ratio (*males*/*males*+*females*) differed between years and was biased towards females when late summer rains were delayed, we used again a GLMM model stating a binomial error. Finally, on the basis of the historical records of the local meteorological station, we analysed the temporal distribution patterns of precipitation recorded for the weevil emergence period over 93 years. All statistical analyses were performed with R packages lme4 and nlme, R Development Core Team.

## 3. Results

Over five study years, we trapped a total of 586 adult weevils. Every year captures were concentrated in a period of one and a half months between 2 September and 19 October—weeks 5 to 12 according to our division. The number of weevils trapped differed between years and was positively related to the total amount of rain recorded between weeks 3 and 10—two weeks before the first and the last weevil emergences (table 1).

**Table 1.** Results from the GLMMs assessing the relationships between: (a) the total number of weevils trapped each year and total amount of rain recorded between weeks 3 and 10 of the study period each year (two weeks before the first and the last weevil emergences). (b) The number of males collected each week at the emergence traps and the number of weeks after the first weevil emergence. (c) The number of females collected each week at the emergence traps and the number of weeks after the first weevil emergence. The first week of the study period started on 4 August and the first weevil emergences were registered on week 5 (starting on 2 September) in all years. All models assume a Poisson distribution of errors and a logit link function. Italicized values indicate significant relationships.

variable	estimate	s.e.	*z*-value	*p*
(a) total number of weevils trapped				
rain recorded between weeks 3 and 10 of the study period	0.005	0.001	4.52	<*0.001*
(b) total number of males trapped				
number of weeks after the first weevil emergence	−0.01	0.02	−0.62	0.53
(c) total number of females trapped				
number of weeks after the first weevil emergence	0.10	0.02	3.90	<*0.001*

**Figure 1.**
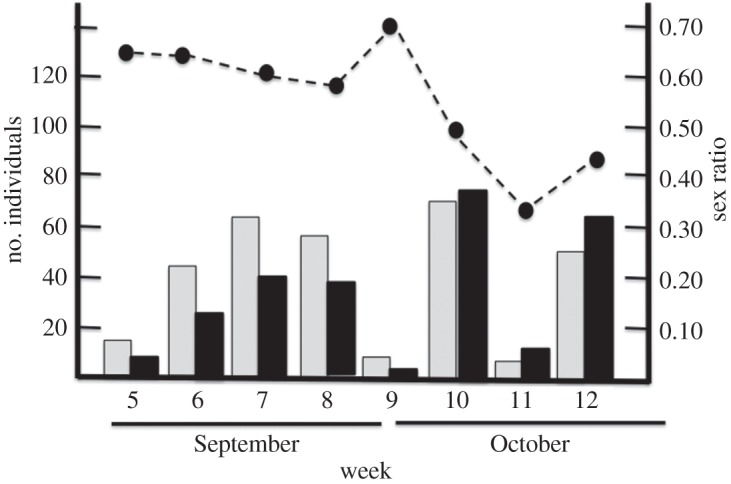
Bar plot depicting the number of males (grey bars) and females (black bars) (left *y*-axis) that emerged in each week of the study period (pooling together the five study years). Weevil emergence started on week 5 (1–7 September) and continued until week 12 (20–26 October). The right *y*-axis shows the temporal variability of the sex ratio [*n* males/(*n* males+*n* females)] (black dots and dashed line) calculated on the number of males and females collected in the emergence traps from weeks 5 to 12.

The number of males collected did not change with the date. By contrast, more females were trapped in the last weeks of the emergence period (table 1 and figure 1) and, consequently, sex ratio decreased significantly over time (table 2 and figure 1). The body size (i.e. elytra length) of the weevils trapped did not changed throughout the emergence period either in males (*F*_1,98_=1.98; *p*=0.16) or females (*F*_1,93_=2.91; *p*=0.09), although in the latter there was a trend towards a larger body size later in the season.

**Table 2.** Results from the GLMMs assessing the relationships between: (a) sex ratio (males/males+females) recorded in the emergence traps each week and the number of weeks after the first weevil emergence. (b) The overall sex ratio recorded each year at the emergence traps and the date of the first rainfall of late summer. The first weevil emergences were registered on the week 5 of the study period (starting on 2 September). The date of the first rainfall of late summer was considered as the date after 4 August with a daily precipitation of at least 1 l m^−2^. All models assume a binomial distribution of errors and a logit link function. Italicized values indicate significant relationships.

variable	estimate	s.e.	*z*-value	*p*
(a) sex ratio				
number of weeks after the beginning of the emergence period	−0.14	0.03	−3.78	<*0.001*
(b) sex ratio				
date of the first rainfall of late summer	−0.02	0.008	−2.4	*0.01*

**Figure 2.**
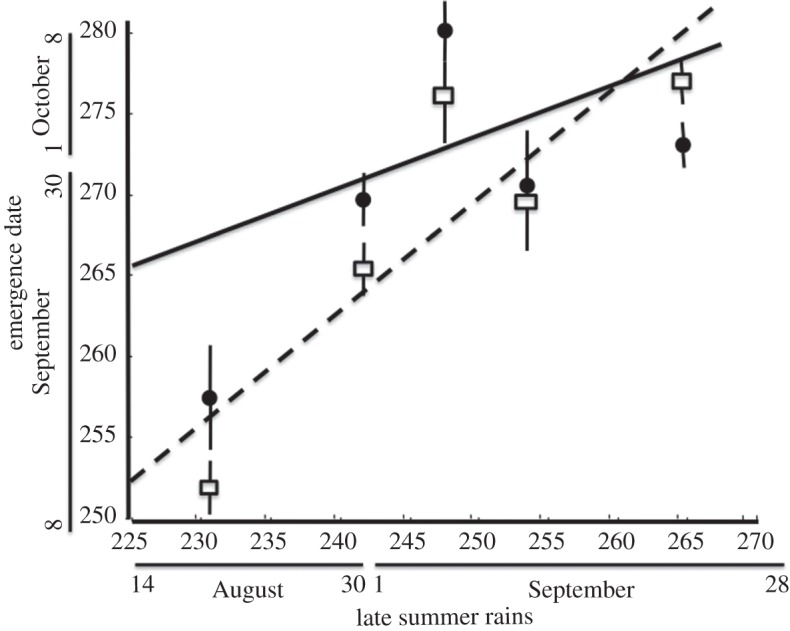
Relationship between the date of the beginning of late summer rains (*x*-axis) and male (white squares) and female (black dots) emergence dates (*y*-axis) in the five study years. The date of the beginning of late summer rains for each year corresponds to the date after 4 August in which at least a daily precipitation of 1 l m^−2^ was recorded. Male and female mean±*s*.*e*. emergence dates for each year were calculated on the mean emergence dates registered for each sex at the emergence traps. The continuous and dashed lines represent the relationship between the date of the beginning of late summer rain and female and male emergence dates, respectively. In all cases, Julian dates are used, 1 January being day 1. On both the *y*-axis and the *x*-axis, the correspondence with the ordinary date format is provided.

**Figure 3.**
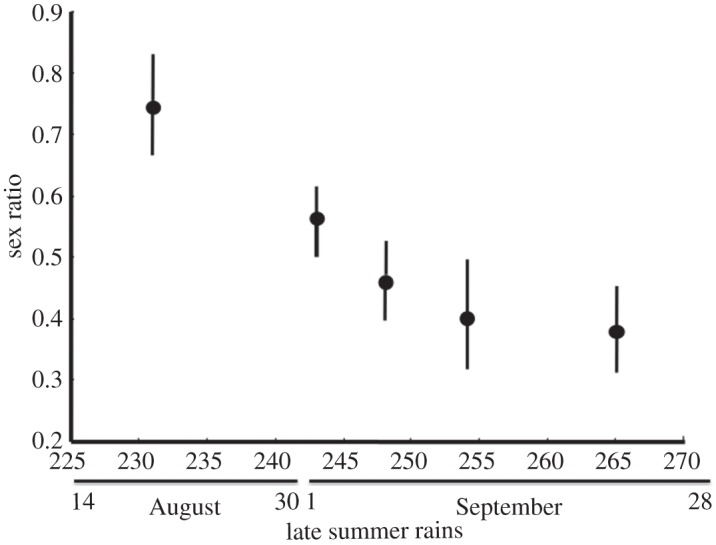
Relationship between the date of the beginning of late summer rains (*x*-axis) and sex ratio [*n* males/(*n* males+*n* females)] in the five study years. The date of the beginning of late summer rains for each year corresponds to the date after 4 August in which at least a daily precipitation of 1 l m^−2^ was recorded. Sex ratio (mean±*s*.*e*.) was calculated on the sex ratios recorded in the emergence traps each year. For the date of the beginning of late summer rains, Julian dates are used, 1 January being day 1; the correspondence with the ordinary date format is provided.

**Figure 4.**
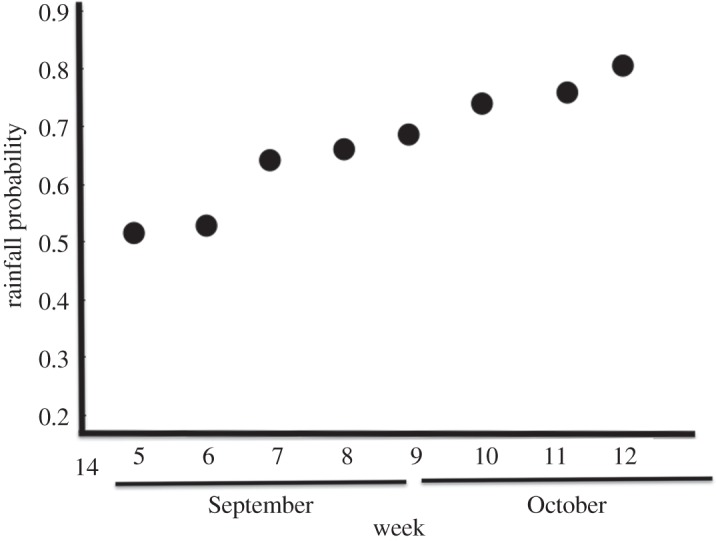
Rainfall probability along the weevil emergence period. The probability (*y*-axis) was calculated on the historical records (1920–2012) of the local meteorological station. It corresponds to the proportion of years in which at least a daily precipitation of 1 l m^−2^ was recorded in the two weeks previous to each of the weeks of the weevil emergence period. Weevil emergence period (*x*-axis) started on week 5 (1–7 September) and continued until week 12 (20–26 October).

The mean emergence date differed between the sexes (*F*_1,175_=7.74; *p*<0.01; figure 2) and was significantly earlier in males than in females (median emergence dates were 26 September and 2 October, respectively). The beginning of late summer rainfall differed a lot between years (more than 30 days; figure 2). Long droughts delayed weevil emergence, as there was a significant positive relationship between weevil emergence date and the beginning of late summer rains (*F*_1,175_=46.76; *p*<0.01; figure 2). However, the effect of the rain timing differed between the sexes (*F*_1,175_=9.22; *p*<0.001): in males, the effect was stronger, early rains triggered a significant advance of emergence date, whereas female emergence was less affected and differed less between years (figure 2). Accordingly, the timing of the late summer rains biased the sex ratio recorded in the emergence traps, which averaged 0.50 pooling all adults captured over the whole study period but differed significantly between years. In those years in which late summer rains started later (i.e. longer droughts), the population sex ratio was biased towards females (table 2 and figure 3). In one of the study years (2011), nonetheless, sex ratio was strongly male-biased (0.74). In 2011, it just rained a little very early in the season and no precipitation was recorded till the end of the weevil emergence period. However, this rain regime is extremely rare; in fact, pooling together the meteorological data from 93 years, we assessed that rainfall probability increased with the date. The likelihood of a daily precipitation over 1 l m^−2^ in the previous two weeks was significantly higher in the last weeks of the weevil emergence period (*F*_1,6_=90.39; *p*<0.0001; figure 4).

## 4. Discussion

This paper is, to the best of our knowledge, the first showing that the timing of precipitation has the potential to affect the sex ratios of some organisms. When late summer rainfall was delayed, the observed population sex ratio was biased towards females. Our data show that the number of females trapped increased significantly in the last weeks of the emergence period. We think that females are thus less vulnerable to longer droughts due to their later emergence from the underground refuges. Males, by contrast, could be ready to emerge earlier, but they might risk dying underground if by then the soil were not soft enough.

Previous reports have shown that weevil emergence is triggered by late summer rainfall [13,14]. This paper supports rainfall constraints, as we collected more weevils in those years in which more rain was recorded over the emergence period. Moreover, in the laboratory, we have checked that weevils reared in plastic containers could not emerge until we added water (R.B., personal observation). Even though we do not have direct field evidence of male-biased mortality caused by dry soil, our data show that males are ready to emerge earlier than females. The effect of the starting date of the late summer rains was stronger in males. When late summer storms were early (i.e. shorter summer droughts), males advanced their emergence proportionally more than females.

The 93-year meteorological record showed that 2011 had unusual rainfall, which coincided with a male-biased sex ratio (0.74). Nonetheless, this was an extremely unusual year because it rained early and no precipitation was recorded for the rest of the weevil emergence period. When this happens, females may probably face difficulties, as the soil will probably be dry again during the peak of their emergence later in the season. In these circumstances, their larger body size and longer rostrum may further complicate emergence [14]. However, the historical meteorological series shows that these conditions are rarely met at our study area. In fact, pooling the data from 93 years, we found that rain probability steadily increased from the beginning to the end of the weevil emergence period.

We have no data on the primary sex ratio, as no laboratory experiments have been conducted to assess whether females have mechanisms to allocate resources to one sex or the other. However, it seems rather unlikely that the patterns reported here result from differential investment in sons and daughters, because females cannot possibly predict the rain timing a year in advance. The unpredictability of future environmental conditions (rainfall or food availability) rules the life histories of acorn *Curcuilo* spp. These insects have a variable length diapause that is considered to have evolved as a bet-hedging strategy to compensate for that unpredictability [17]. Females produce larvae that will emerge in three different years because they cannot predict whether a long drought will kill all her offspring in the following summer [14,17] or whether food availability will be dramatically low due to the inter-annual extreme fluctuations of acorn crops [18]. This risk-spreading strategy maximizes female fitness in the long term due to the lack of reliable clues [17].

If emergence timing has a genetic basis, we could expect a strong selection against early emergence due to the increased risk of mortality when late summer rainfall is delayed. However, there are other evolutionary pressures that may have favoured it. The observed differences in emergence phenology between males and females are characteristic of protandric species, in which females reproduce just after emerging and early males are more likely to copulate than late ones [10]. *Curculio elephas* fits within these characteristics, as females mate and start ovipositing as soon as they climb up the trees [19]. Early emerging males would be thus more likely to be active before the peak of female emergence and could have more chances to mate, unless their phenotype were less preferred by females (e.g. smaller body size). However, this was not the case in *C. elephas*, as male size was not related to emergence date.

The lower total summer precipitation expected in the Mediterranean region according to the climate change models may decrease the population size of species like *C. elephas*, as the amount of precipitation during the emergence period and weevil numbers were strongly related. The forecasted changes in rainfall timing (longer droughts) may, in addition, delay late summer rains and skew sex ratio towards females. Under the current conditions, our results show that these deviations are punctual, as taking the five study years they were compensated and the overall sex ratio for this period was 0.5. However, this balance could be upset if, as predicted, the frequency of dry summers increases [1,2]. The consequences of a female-biased sex ratio for the viability of *C. elephas* populations are probably limited or null. From laboratory experiments in captivity, including paternity assignments using DNA microsatellites, we know that the same male can mate with different females and vice versa (R.B. 2015, unpublished data). Hence, the effective population size will be preserved because, thanks to polyandry, females would very rarely be male limited [20]. Female-biased sex ratio could, however, be detrimental by reducing the population genetic diversity [21], but we lack long-term data to know if a reduction in the number of males of the magnitude observed is enough to provoke it.

## 5. Conclusion

We found that the delay of late summer rainfall biases sex ratio towards females in *C. elephas*, which probably does not risk the viability of its populations. However, the consequences could be worse in insect species with a different adult emergence strategy. In a species with a similar ecology but protogynous (earlier female phenology), a longer drought would provoke a male-biased sex ratio that might reduce the effective population size. In a broader context, this paper supports the importance of keeping a phenological perspective to understand the effects of climate change. In this sense, different studies have previously reported some consequences of climate change on the reproductive phenology of different organisms. For example, it has been demonstrated that shorter and warmer winters and higher temperatures early in spring are advancing bird laying dates or plant flowering in temperate regions [22,23]. Our study provides new data showing that alterations in the rainfall timing may not only affect the reproductive phenology but also the observed population sex ratio. In a biodiversity hotspot like the dry Mediterranean region, marked by a climate with sharp climatic contrasts throughout the year, the question is not only what will happen (higher temperature/less rainfall) but also when it will happen (climate timing).

## Data Availability

Data are available at the Dryad Repository and can be located at http://dx.doi.org/10.5061/dryad.4p6v6.
